# Acute Ecotoxicity and Bioconcentration Tests for Se(IV) in Nile tilapia (*Oreochromis niloticus*)

**DOI:** 10.1021/acsomega.4c06165

**Published:** 2024-11-22

**Authors:** Pedro Henrique da Costa, Nathalia dos Santos Ferreira, Ana Rita de Araujo Nogueira, Eduardo Bessa Azevedo, Mario Henrique Gonzalez

**Affiliations:** † National Institute for Alternative Technologies for Detection, Toxicological Assessment and Removal of Emerging Micropollutants and Radioactives (INCT-DATREM), Department of Chemistry and Environmental Science, São Paulo State University (UNESP), São José do Rio Preto, SP, 15054-000, Brazil; ‡ Environmental Technologies Development Laboratory (LDTAmb), São Carlos Institute of Chemistry, University of São Paulo (USP), São Carlos, SP, 13566-690, Brazil; § Applied Instrumental Analysis Group, Embrapa Pecuária Sudeste, São Carlos, SP, 13560-970, Brazil

## Abstract

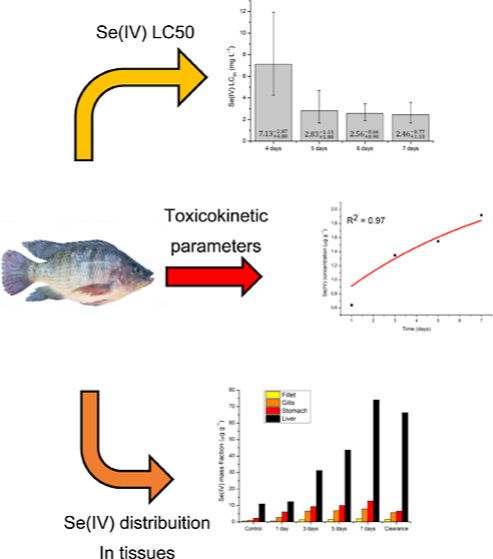

Selenium is one of the most important trace element micronutrients for the global biota, mainly due to its role in protecting against oxidative stress. However, this element can become toxic when present at concentrations slightly higher than those needed for metabolic purposes. It can be transferred through the food chain toward higher trophic levels, with bioaccumulation and biomagnification leading to possible toxicity. This study investigates the bioconcentration and toxicity potential of Se(IV) in Nile tilapia (*Oreochromis niloticus*). After 7 days of exposure, Se concentrations in the fish tissues were in the order: liver ≫ stomach > gills > muscle. In bioconcentration tests, the uptake constant (*k*_a_) ranged from 0.34 to 4.68 mL g^–1^ d^–1^, while the clearance rate constant (*k*_d_) ranged from 0.12 to 0.36 d^–1^. The tissues presented high bioconcentration factors (BCF) ranging from 2.67 to 12.73, demonstrating the ability of Se(IV) to concentrate in muscle, gills, and stomach. Although the data for the liver could not be fitted by the model used, the measured Se(IV) concentrations were approximately six times higher than those found for the stomach, indicating that the *k*_a_, *k*_d_, and BCF values were very high. Estimated LC_50_ values lower than 10 mg L^–1^ suggested that Se(IV) could be considered very toxic to the fish.

## 1 Introduction

Selenium (Se), a nonmetal element belonging to group 16 of the periodic table, is commonly found in nature as anions (selenide, selenite, and selenate), often together with sulfide. Its most stable oxidation numbers are −2, 0, +4, and +6.^1,2^ Inorganic Se species are mainly present in abiotic environments, while organic Se species are predominantly found in biological systems.^3^ Natural sources include volcanic eruptions, rock erosion, soil leaching, and volatilization during biological processes. Industrial and agricultural activities have accelerated the release of Se from geological sources and made it available to fish and other wildlife in aquatic and terrestrial ecosystems worldwide.^4,5^

Se is one of the most important micronutrients, being an essential trace element for microorganisms, plants, animals, and humans. Its main function is to prevent cell damage and it plays important roles in the reproduction, development, and immune systems of both warm-blooded and cold-blooded animals, including humans and fish,^6^ besides being indispensable for the synthesis of selenoproteins, Se has several functions in the response to oxidative stress.^7,8^ It becomes toxic in amounts slightly higher than those required by the organism, causing carcinogenesis, cytotoxicity, genotoxicity, and even teratogenesis.^1,8,9^ Se toxicity can be attributed to two factors. The first is its chemical similarity to sulfur (S), a key component of proteins and linkages between strands of amino acids (ionic disulfide bonds) that are necessary for their tertiary structures and proper function. Studies with mammals and fish have shown that cells cannot differentiate between the two elements (S and Se) during protein synthesis, which results in deformed and dysfunctional enzymes and proteins, causing pathological and teratogenic damage.^10^ The other factor is the oxidative stress caused by the reactions of Se species (mainly the inorganic ones) with thiols, generating reactive oxygen species (ROS) such as the hydroxyl radical (^•^OH), hydrogen peroxide (H_2_O_2_), and the superoxide anion radical (O_2_^•–^). Consequently, the bioaccumulation of this element in tissues can increase the naturally occurring ROS production, damaging biological molecules including lipids, proteins, and DNA.^11^

**Figure 1:**
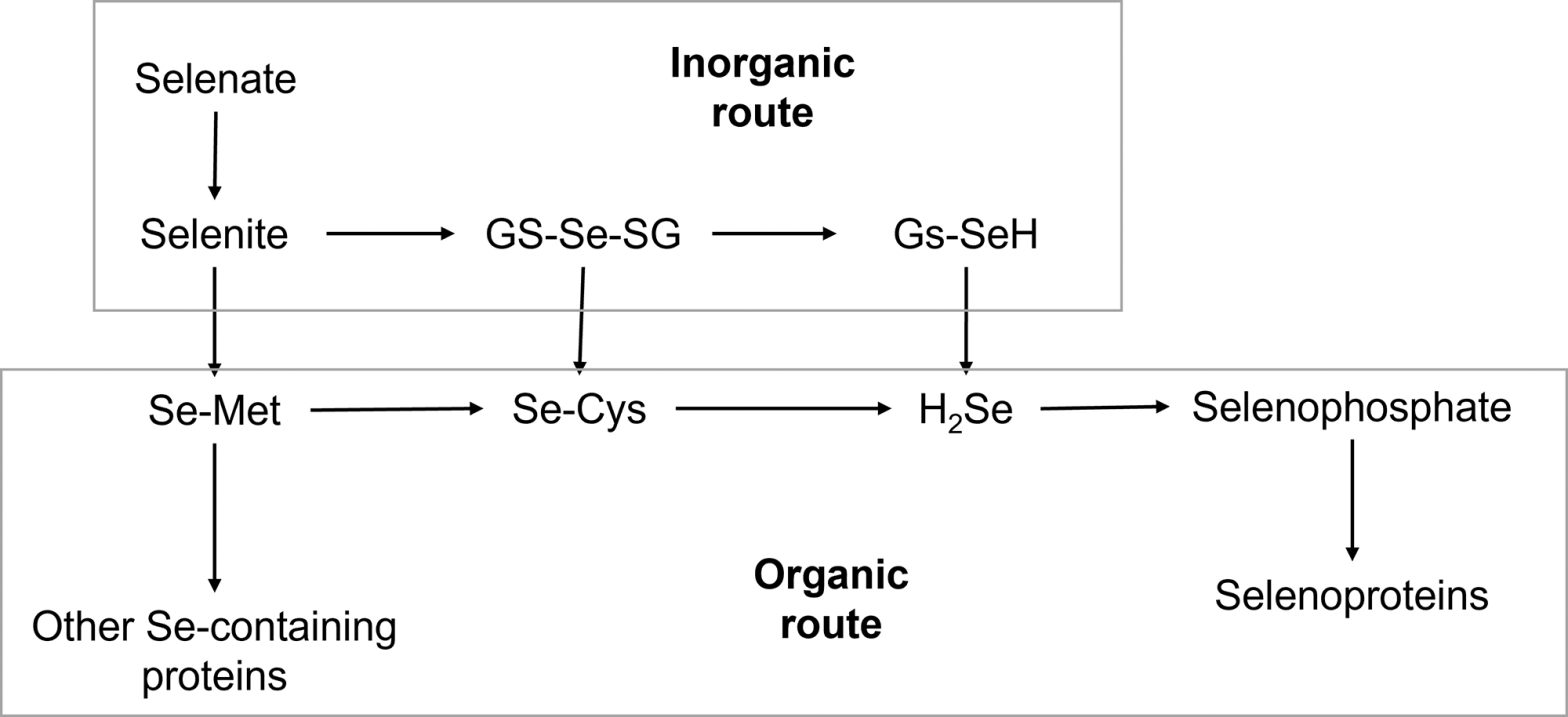
Metabolism of different forms of Se in animals. Modified from Wang et al. (2022). GS–Se–SG: selenodiglutathione; GS–SeH: glutathione selenopersulfide.

The bioavailability of the different forms of Se depends on the retention of absorbed Se and its conversion to the biologically active forms. The main organic forms of Se are selenomethionine (Se–Met) and selenocysteine (Se–Cys), which may originate from the conversion of inorganic forms such as elemental Se, selenate, and selenite, or be assimilated through the food chain, being incorporated into selenoproteins.^12,13^ In fish and other wildlife, the assimilation of water-soluble Se mainly occurs through the gills, epidermis, or gut of the animal.^4^Figure 1 shows the metabolic routes for inorganic and organic Se.

In aquatic environments, animals can accumulate Se by direct contact with water or diet, with the latter being the main route.^7,8^ Primary producers including algae, fungi, and bacteria are responsible for the assimilation of inorganic Se and its conversion to organic forms, which can then be transferred to higher trophic levels through the food chain. Aquatic organisms, other wildlife, and humans can be vulnerable to Se toxicity,^1,5,7^ because Se has the potential to bioaccumulate and biomagnify, as shown by Økelsrud et al. (2016), who reported a trophic magnification factor of 1.29 for Se in two Norwegian lakes, despite low concentrations (22–59 ng L^–1^).

Fish, being relatively long-lived organisms, are often used as bioindicators due to their sensitivity to substances released into the aquatic ecosystem. Pollution can induce many effects, including cellular and biochemical responses, as well as changes in behavior, growth, and reproduction. Se-induced deformities are observed mainly in fish larvae and fry, due to their use of the egg yolk, which contains elevated levels of Se for their development.^14,15^ The most common deformities include lordosis (concave curvature in the lumbar region of the spine), kyphosis (convex curvature in the thoracic region of the spine), and scoliosis (lateral curvature in the spine). There may also be missing or deformed fins, gills, gill covers, and eyes, as well as abnormal head shape and deformed mouth.^14^

Nile tilapia is an excellent candidate for use in biological and ecotoxicological tests, since its characteristics include high tolerance to different environmental conditions, rapid growth, easy reproduction, and omnivorous diet.^11,16^ Given the increased consumption by humans of aquatic animals worldwide (3% increase annually since 1961), together with the simplicity of tilapia cultivation, this aquaculture activity has become one of the most significant commercial fishery industries. Tilapia now ranks second in farmed fish production, with 6.4 million tons produced in 2022.^17^

Several studies have used this fish genus to assess the concentrations of other chemical elements, contributing to its importance as an indicator of environmental pollution. Liao et al. (2003) used Mozambique tilapia (*Oreochromis mossambicus*) as a bioindicator to determine arsenic concentrations in a region of Taiwan with numerous cases of a disease caused by long-term exposure to arsenic. More recently, Rizk et al. (2022) quantified the concentrations of Cd, Zn, Pb, and Cu in the liver and muscle of Nile tilapia (*Oreochromis niloticus*), water, and sediment from a lake in Egypt.

Therefore, the aim of this work was to perform acute ecotoxicity tests to determine the lethal concentration (LC_50_) of Se(IV) in Nile tilapia. Bioconcentration tests were performed to investigate the distribution of Se(IV) in different fish tissues, with estimation of the corresponding toxicokinetic parameters (including absorption and clearance constants) and bioconcentration factors.

## 2 Materials and Methods

### 2.1 Reagents

All the solutions of Se(IV) used in the tests were prepared by successive dilutions of 1000 mg L^–1^ sodium selenite (Na_2_SeO_3_) and concentrated nitric acid (HNO_3_, 65%), both acquired from Sigma-Aldrich (St. Louis, MO, USA).

### 2.2 Test Organisms

Individuals of *O. niloticus*, approximately 8 weeks old and 5 cm in length, free from any visible injuries or illnesses, were donated by the Fisheries Institute of São José do Rio Preto. The individuals (*n* = 30 for the bioconcentration tests and *n* = 42 for the acute ecotoxicity tests) were placed in 500 L tanks kept under constant aeration, with controlled temperature (28 ± 2 °C) and 12 h/12 h light/dark cycle, until the beginning of the tests (around 14 days). Feeding was performed daily, before being interrupted 24 h before the tests to avoid contamination from excrement. All the tests were performed in a static system and were approved by the Animal Use Ethics Committee (CEUA) of São Paulo State University (CEUA protocol #227/2020).

### 2.3 Experimental Procedures

The experimental procedures were established according to the methodologies described by Ferreira et al. (2019), OECD (1992), and USEPA (1996) for acute ecotoxicity tests employing freshwater and saltwater fishes. All the statistical analyses considered a confidence level of 95%.

#### 2.3.1 Bioconcentration Assays

The fish were acclimatized in the laboratory for 2 weeks before the experiments. After acclimatization, they were individually placed in plastic aquariums containing 10 L of 1 mg L^–1^ Se(IV) solution. The tilapia used in this experiment had average length and weight of 16 ± 0.9 cm and 116.7 ± 17.4 g, respectively.

The fish were divided into six groups, each with 5 individuals: a control group, with no exposure to Se(IV); four exposure groups, where the fish were removed after 1, 3, 5, and 7 days of exposure; and a clearance group, where the fish were exposed during 7 days, after which they were transferred to aquariums free from the contaminant, where they remained for another 7 days. After the exposures, the fish were removed and immediately anesthetized and sacrificed by immersion in a 28 mg L^–1^ benzocaine solution. After the fish were confirmed to be dead, their liver, stomach, gill, and muscle tissues were removed and stored (separately) in polypropylene vials in a freezer at −20 °C, to maintain tissue integrity.

#### 2.3.2 Acute Ecotoxicity Tests

Acute ecotoxicity tests were performed to determine the LC_50_ values for the fish exposed to Se(IV) during 1, 2, 3, 4, 5, 6, and 7 days. The LC_50_ is the concentration required to cause the death of 50% of the tested population in a determined exposure period.^22^

The experimental procedure was based on published guidelines for acute toxicity assays involving fish.^21−23^ The tilapia used in this experiment had average length and weight of 14.5 ± 1.3 cm and 103.7 ± 24.3 g, respectively. After the acclimatization period, each fish was individually accommodated in a 10 L aquarium. Six groups were established, each with seven individuals, exposed to 0 (control group), 1, 2, 4, 8, and 10 mg L^–1^ of Se(IV), coded as G1 to G6, respectively. These concentrations were chosen according to OECD (1992) guidelines, not exceeding a geometric factor of 2.2, and Ranzani-Paiva et al. (2011).^24^

During the experiments, fish mortality was recorded every 24 h, up to 7 days of exposure. LC_50_ calculations were based on the trimmed Spearman-Karber method.^25^

### 2.4 Total Se Determination

#### 2.4.1 Sample Preparation

Sample preparation was based on the studies of Gonzalez et al. (2009)^26^ and Oliveira et al. (2017).^27^ Tissues collected for the bioconcentration tests were lyophilized using a freeze-dryer (Liotop L101, Liobras, São Carlos, Brazil) for 7 days at −54 °C, homogenized with a mortar and pestle, and submitted to microwave-assisted acid digestion (MW-AD) (Multiwave 3000, Anton Paar GmbH, Austria). The microwave operational parameters were 1100 W at 200 °C, for 20 min, followed by a cooling step of 10 min.

#### 2.4.2 Analyses by Inductively Coupled Plasma Mass Spectrometry

For Se determination, the samples were analyzed using an inductively coupled plasma mass spectrometry (ICP–MS) system (NexION 300X, PerkinElmer, Shelton, CT, USA) equipped with a Meinhard concentric nebulizer, a cyclonic nebulization chamber, and a quartz torch with quartz injector tube (2.0 mm i.d.). The nebulizer gas flow rate, torch alignment, and quadrupole voltage were adjusted according to the manufacturer’s recommendations. The radiofrequency (RF) power was set to 1600 W and the analyses were performed in triplicate. The instrumental parameters were optimized before the analyses. Se calibration curves were obtained by diluting a stock 1000 mg L^–1^ Se(IV) solution in 1% HNO_3_. The method precision was evaluated using certified reference materials (CRMs) of trace elements in natural water (SRM 1640a, National Institute of Standards and Technology, NIST, Gaithersburg, Maryland, USA), dogfish liver (DOLT-5, National Research Council, NRC, Ottawa, Ontario, Canada), and lobster hepatopancreas (TORT-2, National Research Council, NRC, Ottawa, Ontario, Canada). Total Se concentrations were determined using a mass/charge ratio (*m*/*z*) of 82, in kinetic energy discrimination (KED) mode, with helium (He) as the collision gas at a flow rate 1.5 mL min^–1^. It was necessary to use the collision cell due to the interference caused by the argon gas dimer that has the same *m*/*z* as the most abundant isotope of Se (*m*/*z* 80). The ICP–MS instrumental conditions are provided in Table 1.

**Table 1:** Instrumental and Method Parameters for the ICP–MS Analyses

instrumental parameters	
radiofrequency power	1600 W
plasma gas flow rate	18 L min^–1^
auxiliary gas flow rate	1.2 L min^–1^
nebulizer gas flow rate	1.0 mL min^–1^
sample uptake rate	0.7 mL min^–1^
**method parameters**	
scans per reading	50
readings per replicate	1
replicates	3
dwell time	25 s
analytical calibration range	0.1–15 μg mL^–1^

#### 2.4.3 Data Analysis

The accumulation of toxic pollutants in aquatic organisms such as fish occurs according to several routes, considering the equilibrium concentrations in water and in the aquatic organism. The environmental risk assessment for Se (IV) was performed using the first order compartmentalization model, which assumes that the clearance rate after exposure to a contaminant is directly proportional to the concentration of the contaminant in the fish tissue.^28^

The pollutant accumulation capacity was determined for the fish tissues using the bioconcentration factor (BCF),^18−20^ considered as the ratio between the concentration of the contaminant in the fish tissue (*C*_f_, μg g^–1^) and its concentration in the water at equilibrium (*C*_w_, μg g^–1^), as given by eq 1.
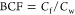
1

The kinetic parameters for absorption and clearance (*k*_a_ and *k*_d_, respectively) were determined using eq 2,^29^ where *C*(*t*) is the time-dependent Se(IV) concentration in the tilapia tissues (μg g^–1^), *k*_a_ is the tissue-specific absorption constant (mL g^–1^ d^–1^), *k*_d_ is the clearance constant (d^–1^), *t* is the exposure period (days), and *C*_w_ is the Se concentration in the water (μg mL^–1^). Nonlinear optimization was used for estimating *k*_a_ and *k*_d_, using the Solver tool of Excel 2016, according to the generalized reduced gradient method.
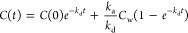
2

The tissue-specific BCF was calculated as the ratio between *k*_a_ and *k*_d_, representing the net capacity resulting from the competition between the absorption and clearance processes.^2^

### 2.5 Statistical Analysis

Statistical analysis was performed for the different tissues, with at least three replicates for each day of exposure, employing Jamovi v. 2.3.28 software (Jamovi, Sidney, NSW, Australia). The data were tested for normality using the Shapiro–Wilk test. For the types of tissues and times of exposure, comparisons were performed using the Kruskal–Wallis test and the Dwass-Steel-Critchlow-Fligner multiple comparisons posthoc test. Graphs were produced using OriginPro v. 8.5 software (OriginLab, Northampton, MA, USA). The statistical significance level considered for all the tests was *p* < 0.05.

## 3 Results and Discussion

### 3.1 Bioconcentration Tests

**Figure 2:**
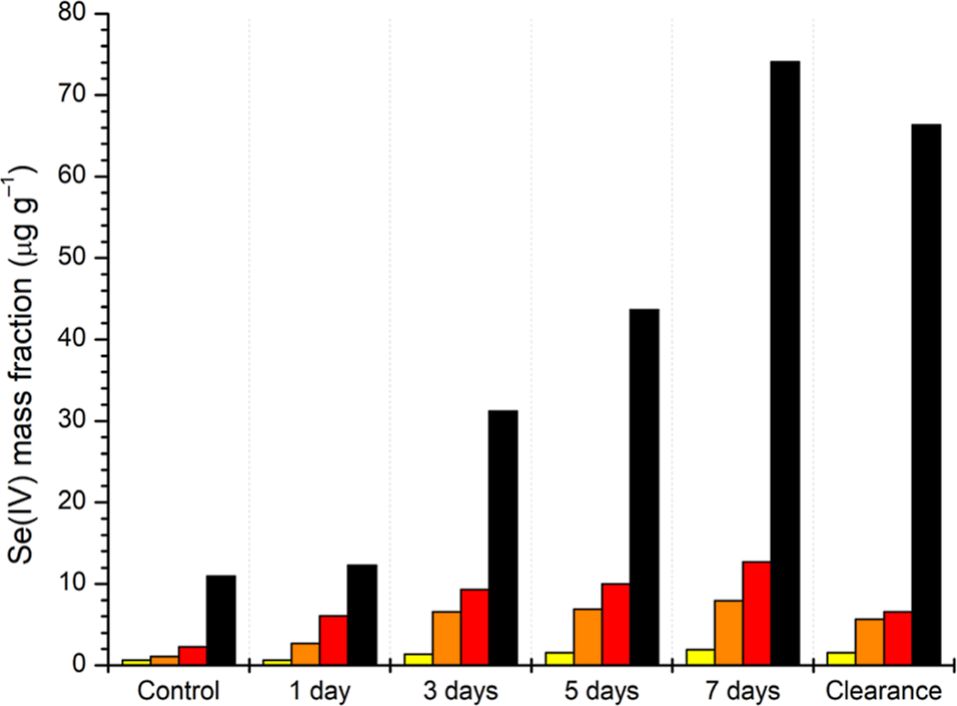
Se(IV) distributions (μg g^–1^) in different fish tissues during the bioconcentration and clearance tests: “muscle (■-yellow), gills (■-orange), stomach (■-red), and liver (■-black)” to “muscle (■), gills (■), stomach (■), and liver (■)”.

Bioconcentration tests performed with different tissues can be used to elucidate the metabolization capacity of aquatic organisms, as well as ecotoxicological potentials. In this work, Se(IV) was selected, because it is about three times more toxic than Se(VI) (Ranzani-Paiva et al., 2011).^24^ The highest level of Se(IV) was found in the liver, followed by the stomach, gills, and muscle. Figure 2 shows the distributions of Se(IV) during 7 days of exposure and then after 7 days of clearance. Similar distribution profiles of Se(IV) for the same tissues, except the stomach, were observed by Li et al. (2008).^30^ The results showed the far greater capacity of the liver to bioconcentrate Se(IV), compared to the other tissues studied, with concentrations up to six times higher, compared to the stomach, which presented the second-highest bioconcentration capacity. After 7 days of exposure, the Se(IV) concentrations in the liver, stomach, gills, and muscle were approximately 74, 13, 8, and 2 μg g^–1^, respectively.

One of the possible reasons for the high levels of Se(IV) observed in the liver is that inorganic Se is predominantly accumulated there for the synthesis of selenoproteins. However, although it presents a greater tendency for the concentration of inorganic Se, the liver is not the only final storage site for Se in fish, since this organ only contributes a small proportion of the whole-body Se, while the skeletal muscle has the highest contribution.^12^Figure 2 shows the Se(IV) mass fractions in different tilapia tissues after the bioconcentration experiment. The order of the Se mass fractions in the tilapia tissues after 7 days of exposure was: liver ≫ stomach > gills > muscle. The amount of the contaminant increased during the 7 day exposure period, followed by a decrease after the 7 day clearance period.

#### 3.1.1 Statistical Analysis

**Figure 3:**
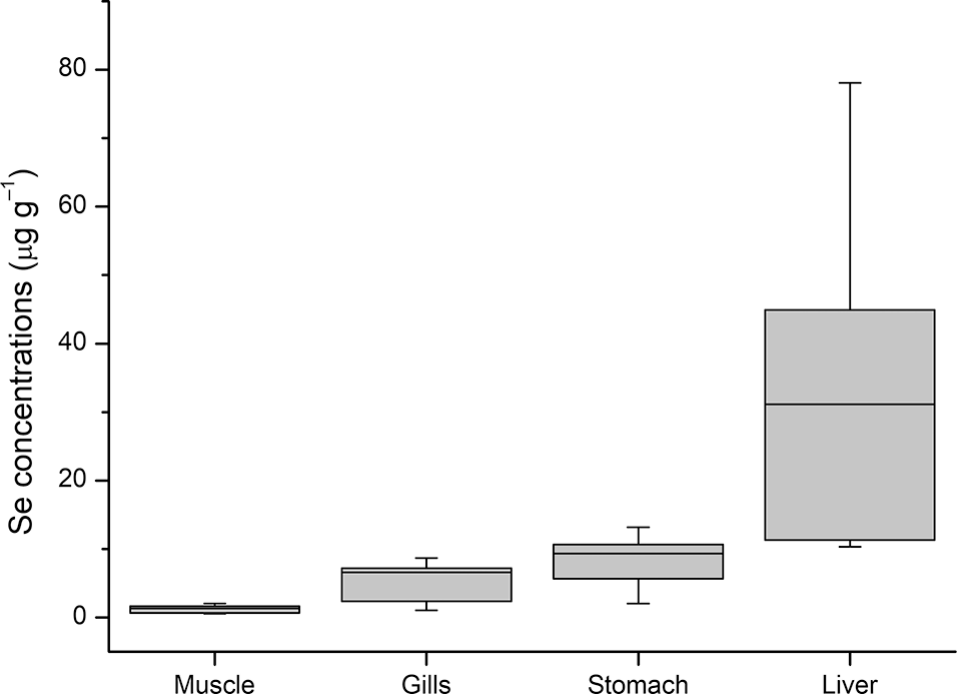
Box plot of the Se(IV) concentrations accumulated in the *O. niloticus* tissues.

First, the Shapiro–Wilk test was applied to evaluate the normality of the data for the different tissues. The calculated *p*-values were 0.074, 0.013, 0.153, and 0.019 for muscle, gills, stomach, and liver, respectively. This indicated that the data for the gills and liver were not normally distributed (*p* < 0.05). Consequently, the Se(IV) concentration data were represented using a box plot (Figure 3).

The data shown in Figure 3 suggested that the bioconcentration behaviors of the muscle and liver were markedly different, while those of the gills and stomach were similar. To confirm this, the Kruskal–Wallis test was applied, due to the nonparametric nature of the data. The *p*-value obtained was <0.001, confirming that there was a statistical difference among the tissues. To determine which tissues differed from each other, the Dwass-Steel-Critchlow-Fligner (DSCF) multiple comparisons test was used as a posthoc test. This revealed that the bioconcentration profiles of the gills and stomach were statistically the same, despite these tissues having cellular and structural dissimilarities, while the bioconcentration profiles of the muscle and liver differed. Table 2 shows the calculated *p*-values. It is possible that the use of other organs, such as intestine, heart, or kidneys, instead of stomach/gills, could assist in further elucidating the toxic behavior of Se(IV).

**Table 2:** DSCF Multiple Comparisons Test Data

		*p*-value
muscle	gills	0.002
muscle	stomach	<0.001
muscle	liver	<0.001
gills	stomach	**0.142**
gills	liver	<0.001
stomach	liver	<0.001

#### 3.1.2 Toxicokinetic Parameters

**Figure 4:**
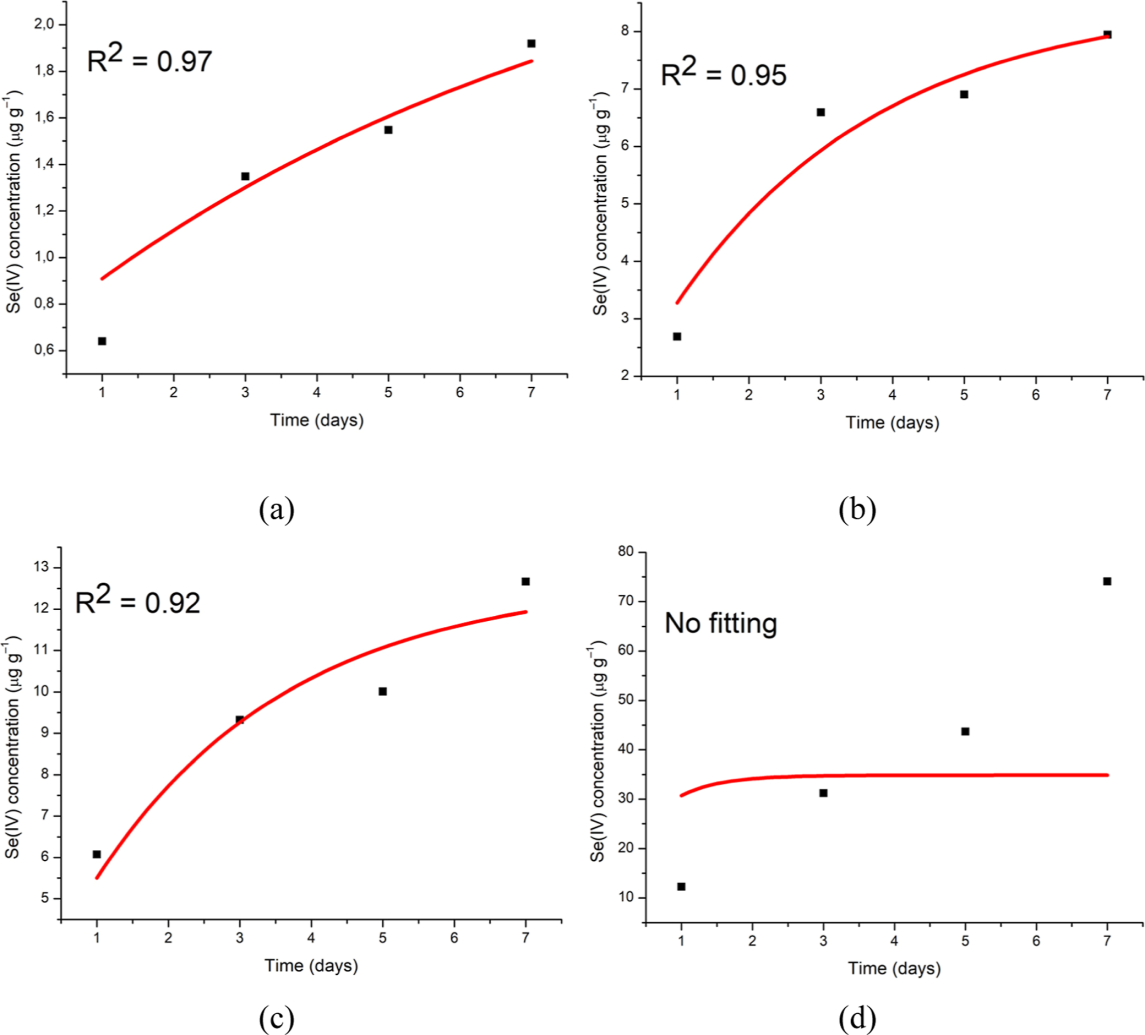
Comparison between measured (■) and modeled (−) Se(IV) concentrations in each tissue: (a) muscle, (b) gills, (c) stomach, and (d) liver.

Figure 4 shows the results of fitting eq 2 to the experimental bioconcentration data. Significant determination coefficients were obtained for the stomach, muscle, and gills (*R*^2^ = 0.92–0.97, *p* < 0.05), indicating satisfactory fitting by the model. The model did not provide a good fit to the liver data, presenting a linear behavior. This could have been due to the high bioconcentration capacity of the liver, with a longer exposure period being required for tissue saturation.

Table 3 presents the toxicokinetic parameters obtained for Se(IV) in the tilapia muscle, gill, and stomach tissues. The high values obtained could be attributed to the fact that Se is an essential nutrient used by several organisms in proteins such as glutathione peroxidase (GPx), thioredoxin reductase (TrxR), and deiodinase iodothyronine (ID) to regulate the oxidative microenvironment.^31^ For comparison purposes, the toxicokinetic parameter values for As(III), reported by Ferreira et al. (2019), were lower than the values obtained here, reflecting the importance of Se in biological organisms.

**Table 3:** Toxicokinetic Parameters for Se(IV) and As(III) in Tilapia Tissues

tissue	***k***_***a***_ (mL g^–1^ d^–1^)		***k***_***d***_ (d^–1^)		BCF (mL g^–1^)	
	Se(IV)	As(III)^a^	Se(IV)	As(III)^a^	Se(IV)	As(III)^a^
muscle	0.34	0.20	0.12	1.15	2.67	0.2
gills	2.98	0.06	0.34	0.07	8.57	0.8
stomach	4.68	0.12	0.36	0.08	12.73	1.5

a Data from Ferreira et al. (2019).

Although it was not possible to estimate the toxicokinetic parameters for the liver, the absolute Se(IV) concentrations obtained for this tissue (Figures 1 and 2) were approximately six times higher than those found for the stomach. Therefore, the *k*_a_, *k*_d_, and BCF values were far higher than those reported in Table 3.

### 3.2 Acute Ecotoxicity Test

The average lethal concentration (LC_50_) value is the amount of a chemical species capable of causing the death of 50% of the affected population. To calculate this concentration, there must be death events in the population above and below 50% of the number of individuals used in each group (*n* = 7, in the present experiment).

Table 4 presents the cumulative percentages of deaths that occurred during the acute ecotoxicity tests. Death events were counted every 24 h during the exposure period and each fish represented 14.3% of the total. No deaths in any group were observed in the first 2 days.

**Table 4:** Cumulative Percentages of Deaths Every 24 h in the Acute Ecotoxicity Tests

group	days of exposure				
	3	4	5	6	7
G1					
G2					14.3
G3		14.3	28.6	28.6	28.6
G4		14.3	71.5	85.8	85.6
G5	28.6	57.2	71.5	100	100
G6	28.6	28.6	42.9	85.8	100

**Figure 5:**
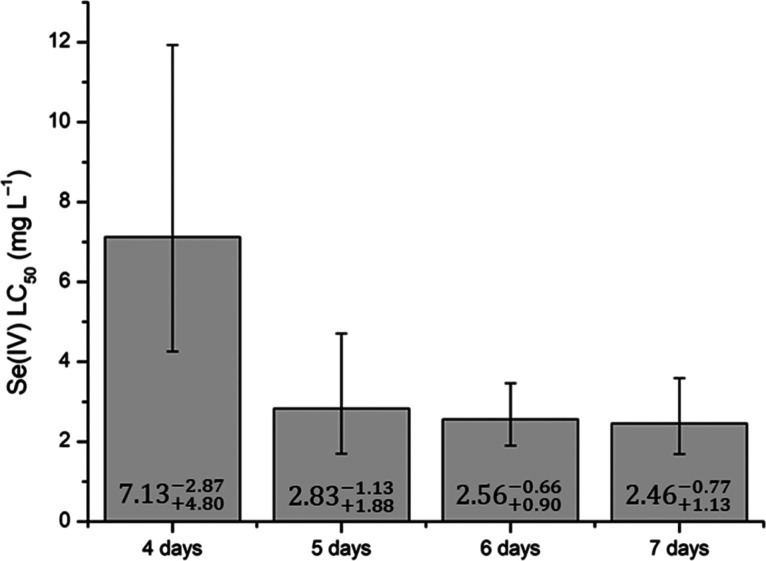
Lethal concentration (LC_50_) values calculated for Se(IV).

The calculated LC_50_ values are shown in Figure 5. Due to the small number of deaths in the groups during the first 3 days of testing, it was not possible to determine the LC_50_ for this exposure period. The LC_50_ values obtained for 5, 6, and 7 days were similar, with death events occurring when the Se(IV) concentration in the medium exceeded 3.00 mg L^–1^. However, it could be seen that the LC_50_ decreased as the exposure time increased, due to the increase in contaminant absorption over time.

According to the toxicity categories for fish, provided by the book “A Textbook of Modern Toxicology”^32−34^ (Table 5), the LC_50_ values determined here for sodium selenite, in the concentration range between 1 and 10 mg L^–1^, indicated that the chemical compound was very toxic to the fish.

**Table 5:** Ranking of the Acute Toxicity of Chemicals to Fish^a^

fish LC_50_ (mg L^–1^)	toxicity rank
>100	relatively nontoxic
10–100	moderately toxic
1–10	very toxic
<1	extremely toxic

a Adapted from Leblanc and Buchwalter (2010).

The LC_50_ values for Se(IV) reported in the literature vary according to the species and the size of the organism. Ma et al. (2018) and Takayanagi (2001) showed that the concentrations capable of causing the death of 50% of the population after 96 h of exposure were 3.62 mg L^–1^ (*Pseudorasbora parva*) and 11.5 mg L^–1^ (*Pargus major*), respectively. Therefore, the LC_50_ value obtained here, for an exposure period of 96 h, was within the range reported previously. Another relevant study was that of Ranzani-Paiva et al. (2011),^24^ who determined the LC_50_ of Se(IV) for *O. niloticus* fingerlings, with a value of 4.42 mg L^–1^ obtained for an exposure period of 96 h. The size of the fish used could explain the lower LC_50_, since fish fingerlings are more sensitive to the concentrations of contaminants in the medium.

## 4 Conclusions

Se(IV) bioconcentration tests showed that the liver plays an important role in the metabolization of this element in tilapia. The toxicokinetic parameters (*k*_a_ and *k*_d_) and BCF showed that all the studied tissues (muscle, gills, stomach, and liver) were capable of accumulating Se(IV), which could be ascribed, at least in part, to the natural presence of Se in the tilapia organism, since this element is incorporated into proteins that provide defense against oxidant agents.

The bioconcentration capacity of the tissues followed the order: muscle < gills < stomach ≪ liver. This finding leads to two conclusions:

Whenever possible, the liver of the organism should not be disregarded in bioconcentration tests involving Se(IV), since it may be the organ/tissue that corresponds to the worst-case scenario, especially if biomagnification is an issue.

The muscle showed the lowest bioconcentration potential, among the tissues studied. Hence, since tilapia is a fish commonly present in the diet of humans, there seems to be a relatively low risk of any significant Se contamination arising from the consumption of this type of fish.

Acute ecotoxicity tests showed that the calculated 96 h LC_50_ values, as well as those calculated for different exposure periods, were within the range from 1 to 10 mg L^–1^, indicating that sodium selenite could be considered a very toxic compound.

To date, most of the studies concerning the various forms of Se in organisms have focused on its antagonistic effects against contamination by potentially toxic elements, such as mercury, or the effects of selenium insufficiency, given that it is an essential micronutrient. However, bioconcentration and acute toxicity tests must be performed to assess the potential of Se as a contaminant, since it becomes toxic at concentrations slightly higher than those supplied by the diet to ensure normal metabolism. Considering that Se is commonly added to the diets of farmed fish, their increasing worldwide exportation and consumption may pose a real threat to the health of human beings.

The main contributions of this study are that it addresses the lack of information concerning Se as a contaminant, elucidating the bioconcentration behavior of Se in important organs and determining the lethal concentration for sodium selenite. Future studies will focus on the toxicity of different sources of Se, as well as its antagonist effects in the presence of other contaminants such as arsenic (As) or lead (Pb).
